# Hemolytic Uremic Syndrome: Late Renal Injury and Changing Incidence—A Single Centre Experience in Canada

**DOI:** 10.6064/2012/341860

**Published:** 2012-12-31

**Authors:** Pierre Robitaille, Marie-José Clermont, Aïcha Mérouani, Véronique Phan, Anne-Laure Lapeyraque

**Affiliations:** Division of Nephrology, Department of Pediatrics and Sainte-Justine Hospital (CHU Sainte-Justine), University of Montreal, 3175 Côte Ste-Catherine, Montreal, QC, Canada H3T 1C5

## Abstract

*Aims*. To assess trends in the incidence of pediatric diarrhea-associated hemolytic uremic syndrome (D^+^ HUS) and document long-term renal sequelae. *Methods*. We conducted a retrospective cohort study of children with D^+^ HUS admitted to a tertiary care pediatric hospital in Montreal, Canada, from 1976 to 2010. In 2010, we recontacted patients admitted before 2000. *Results*. Of 337 cases, median age at presentation was 3.01 years (range 0.4–14). Yearly incidence peaked in 1988 and 1994-95, returning to near-1977 levels since 2003. Twelve patients (3.6%) died and 19 (5.6%) experienced long-term renal failure. Almost half (47%) The patients required dialysis. Need for dialysis was the best predictor of renal sequelae, accounting for 100% of severe complications. Of children followed ≥1 year (*n* = 199, mean follow-up 8.20 ± 6.78 years), 19 had severe and 18 mild-to-moderate kidney injury, a total sequelae rate, of 18.6%. Ten years or more after-HUS (*n* = 85, mean follow-up 15.4 ± 5.32 years), 8 (9.4%) patients demonstrated serious complications and 22 (25.9%) mild-to-moderate, including 14 (16%) microalbuminuria: total sequelae, 35.3%. *Conclusions*. Patients with D^+^ HUS should be monitored at least 5 years, including microalbuminuria testing, especially if dialysis was required. The cause of the declining incidence of D^+^HUS is elusive. However, conceivably, improved public health education may have played an important role in the prevention of food-borne disease.

## 1. Introduction

Hemolytic uremic syndrome (HUS) is the most frequent cause of acute kidney injury in children. Approximately 5% of children with HUS die within the acute phase of the disease, with early studies reporting a mortality rate as high as 21% [1, 2]. As many as 25% of HUS survivors sustain long-term renal sequelae [3]. 

HUS is a systemic microangiopathy characterized by acute hemolytic anemia, thrombocytopenia, and renal failure [4]. In the classic or typical form, it is preceded by a prodrome of bloody diarrhea and gastroenteritis (D^+^ HUS) due to infection with strains of *Escherichia coli* (*E. coli*) that produce Shiga-like toxin, also known as verotoxin [3]. Shiga toxin-producing *E. coli* (STEC), or verotoxin-producing *E. coli* (VTEC), such as *E. coli* O157:H7 have been systematically recovered from stools of patients with D^+^ HUS since the mid-1980s, when this association was clearly established [5]. An atypical form of HUS has also been described. Accounting for 5–10% of pediatric HUS cases, the atypical form HUS is due to a variety of causes, including complement pathway abnormalities [6]. 

The percentage of HUS patients presenting with chronic renal complications varies considerably from one series to another, in part because cohorts were of variable sizes, large percentages of patients were lost to followup, or duration of the observation period was too short. A more accurate estimate of the long-term renal outcome is thus of paramount importance for appropriately guiding patient followup and counselling. Furthermore, our overall experience seemed to suggest a lower incidence of HUS in recent years, a phenomenon perhaps explainable by increased public health campaigns. To that end, we analyzed the experience of a large, tertiary care academic pediatric hospital in Montreal, Canada. 

## 2. Patients and Methods

### 2.1. Study Design

We conducted a retrospective cohort study of children aged 0–18 years treated at Sainte-Justine Hospital (CHU Sainte-Justine) for D^+^ HUS from 1976 to 2010. We examined each patient chart for survival as well as short- and long-term sequelae. CHU Sainte-Justine is a 450-bed tertiary care teaching hospital in Montreal, one of the largest pediatric centres in Canada, with a catchment area that covers roughly 50% of the Quebec patient population. In addition, the Sainte-Justine outpatient nephrology clinic provides long-term followup when desired. The approval for the study was obtained from the institution's Research Ethics Board, and written consent was provided by the parents. 

Inclusion criteria for postinfectious HUS were the following a prodrome of gastroenteritis with hemorrhagic colitis followed by microangiopathic hemolytic anemia, thrombocytopenia, and acute nephropathy (D^+^ HUS). 

Medical charts of surviving patients were examined for the presence of microalbuminuria, proteinuria, systemic hypertension and renal function. Microalbuminuria was defined as an albumin/creatinine ratio >2.0 mg/mmol [7], preferably tested on a morning sample. Proteinuria was defined as ≥1+ on dipstick analysis [8] or a urinary albumin/creatinine ratio >30 mg/mmol [9]. Glomerular filtration rate (eGFR) was estimated using the Schwartz formula for creatinine clearance; a creatinine clearance rate <80 mL/min per 1.73 m^2^ was considered abnormal [10, 11]. This method was chosen as a convenient representation of renal function notwithstanding a number of inherent limitations [12]. Hypertension was defined as arterial blood pressure higher than the 95th percentile for age and gender of the child [13], generally corresponding to 130/80 mm Hg in adults.

In 2010, we contacted the subset of patients who had presented with D^+^ HUS at least 10 years prior, and asked them to come in for reevaluation of their renal status, including measurements of blood pressure as well as determinations of GFR, proteinuria, and microalbuminuria from blood and urine samples. An albumin/creatinine ratio greater than 2.0 mg/mmol was considered abnormal [4].

### 2.2. Statistical Analysis

Statistical analyses of data, including averages, standard deviations (SD), *P* values, and chi-square tests were performed using GraphPad, InStat 3 software (GraphPad Software, Inc., La Jolla, CA, USA). Unpaired *t*-tests, paired *t*-tests, or the Mann-Whitney test were used whenever appropriate. Trends in yearly incidence of D^+^ HUS were determined with segmented regression analysis using SAS 9.1 software for Windows (SAS Institute Inc., Cary, NC, USA).

## 3. Results

### 3.1. Patient Characteristics

**Figure 1:**
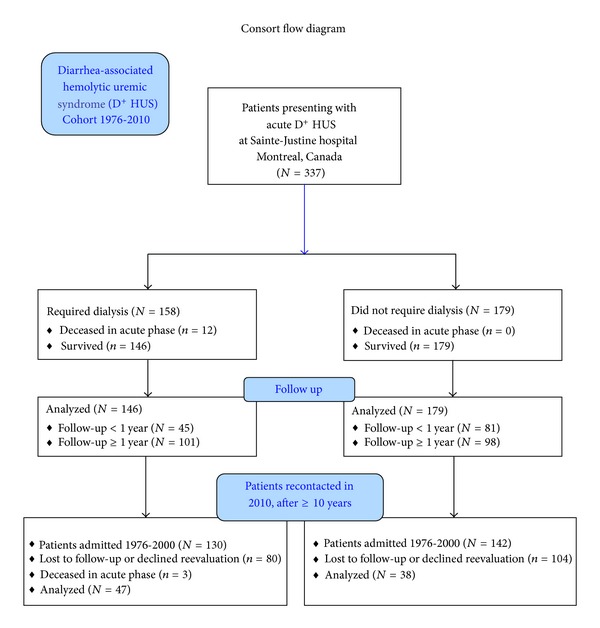
Retrospective cohort of patients admitted to hospital for acute diarrhea-associated hemolytic uremic syndrome (D^+^ HUS) from 1976 to 2010.

Our retrospective cohort consisted of 337 children admitted at our institution between 1976 and 2010 for D^+^ HUS (Figure 1). The majority (96%) were Caucasian. Over half (54%) of the patients were 3 years of age or younger at the time of admission, and 81% were aged 6 years or less. There was no significant difference in the percentage of boys and girls (*P* = 0.1).

### 3.2. Incidence

**Figure 2:**
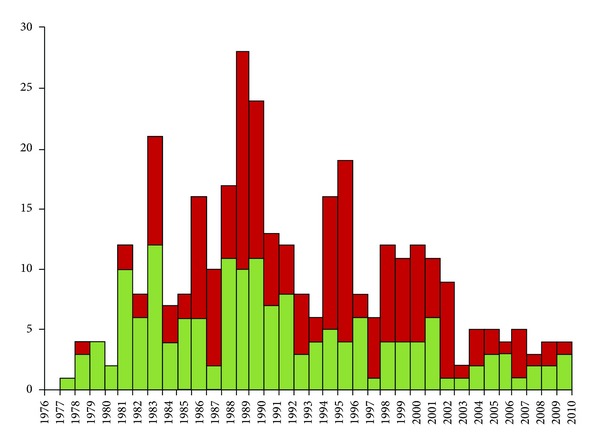
Yearly incidence of acute diarrhea-associated hemolytic uremic syndrome, from 1976 to 2010. Red bars indicate total number of patients. Green bars represent number of patients requiring dialysis.

The yearly incidence of D^+^ HUS varied significantly over time (Figure 2). In the late 70s, less than 5 cases were diagnosed per year. In the 80s, the incidence rose. The number of new patients peaked at 21 in 1982, decreased, and then peaked again at 28 and 24 in 1988 and 1989, respectively. Another but lesser peak was observed in 1994-95, at 16 to19. Since 2003, the number of new cases has returned to 5 or fewer per year. Overall, the incidence of reported cases has decreased significantly since 1988 (*P* < 0.001).

### 3.3. Outcomes

#### 3.3.1. Short-Term Outcome

Since no specific treatment was available for D^+^ HUS until 2010, only basic supportive measures were used in the acute phase. These included adequate fluid intake, plasma volume expansion, the use of antihypertensive medication, and the administration of loop diuretics at the initial phase of the acute illness in an attempt to obviate the need for replacement therapy [1, 14]. Renal replacement therapy was instituted in patients exhibiting oliguria (<240 mL/m^2^ per day) for at least two consecutive days or anuria (<15 mL per day) for 24 hours or more. Patients remained in the intensive care unit as long as replacement therapy was required.

**Figure 3:**
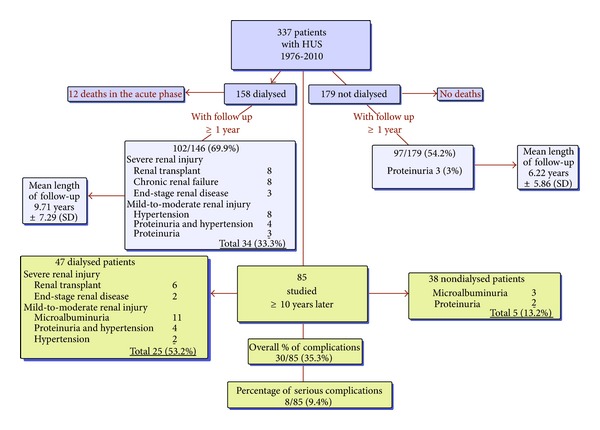
Patient outcomes.

Twelve patients died in the acute phase of HUS. All had multiorgan failure and severe neurological involvement and had required renal replacement therapy (Figures 1 and 3). Of the 325 children who survived the acute phase, 146 (44.9%) required renal replacement therapy, of whom 139 (95%) received peritoneal dialysis, the most frequently used modality. The remaining 7 children required hemodialysis owing to the severity of gastrointestinal involvement, including bowel perforation, and/or pancreatitis. 

#### 3.3.2. Follow-Up ≥1 Year

The difference in overall length of followup between patients necessitating dialysis, and those who did not need dialysis were statistically significant, mean ± SD of 9.71 ± 7.29 versus 6.22 ± 5.86 years, respectively; *P* < 0.0094 (Figure 2). 

We compared the 199 (61%) patients with long-term follow-up (≥1 year) to those with shorter-term follow-up (<1 year) (Table 1). There were no significant differences in age at HUS presentation or in white blood cell count. However, dialyzed patients with long-term followup had received significantly more days of dialysis (9.23 ± 6.04 versus 6.40 ± 4.83 days, resp.; *P* < 0.001). 

Of the 102 dialyzed patients with long-term followup, 34 (33.3%) had sustained permanent kidney damage (Figure 3). Eight required a kidney transplant, 3 had end-stage renal disease (GFR < 15 mL/mL/1.73 m^2^), 8 chronic renal failure (GFR 15–59 mL/mL/1.73 m^2^), and 15 less-severe complications with a normal GFR. By the end of the acute phase, none of the 19 patients with severe kidney damage had recovered normal renal function. Two required dialysis for more than one month, after which time they were only temporarily dialysis-free. 

There were no deaths in the group not requiring dialysis. None developed permanent kidney damage. Only 3 of the 97 patients followed up for ≥1 year had mild-to-moderate proteinuria (<2 g/day), and none were hypertensive.

To summarize, 19 of 199 (9.5%) patients with followup of at least one year had severe renal complications while 15 had minor complications, for a total of 34 cases (17%) with renal sequelae after surviving HUS. Thus as many as 83% of this cohort appeared to be free of renal injury after an average followup of 8.24 ± 6.80 years. It is noteworthy that peripheral white blood cell count was not associated with the need for dialysis during the acute phase (16.76 ± 7.58 versus 16.62 ± 8.68 × 10^9^/L, resp.). 

#### 3.3.3. Follow-Up ≥10 Years

Of the 274 children admitted between 1976 and 2000, 85 (32%) agreed to come in for reevaluation of their renal status in 2010. The time elapsed between the acute episode and reevaluation thus varied from 10 to 34 years, with a mean of 15.7 ± 4.88 years. Approximately one-third of participants were readily accessible as they had never ceased receiving specialized medical care. The remainder was located by various means, including internet sites such as *Find a person*, *Facebook*, or the like. 

Approximately half of participants (47/85) had received dialysis, 25 of whom suffered permanent renal injury, including 6 with kidney transplants and 2 with end-stage renal disease (Figure 3). Less-severe sequelae included microalbuminuria in 11 participants, mild-to-moderate proteinuria and hypertension in 4, and proteinuria alone (<2 g/day) in 2. Their mean follow-up (14.39 ± 5.1 years) and dialysis (13.14 ± 6.17 days) were significantly longer than for the 22 participants free of renal involvement (17.92 ± 5.82 years, and 8.0 ± 5.18 days, resp.); *P* = 0.0037.

Of the 38 participants who had not required dialysis, none sustained severe renal complications, over an average of 13.95 ± 3.91 years. Proteinuria was detected in 2 patients and microalbuminuria in 3. Thus, 33 (86.8%) of nondialyzed patients appeared to be free of renal injury.

Taken together, 55 of 85 cases followed for well over than 10 years on average were spared renal injury secondary to HUS. Of the remainder, 8 (9.4%) patients sustained serious complications while 22 (25.9%) had signs of less-severe renal damage.

## 4. Discussion

In this retrospective cohort study of classic HUS in Montreal, Canada, we observed a yearly incidence of D^+^ HUS decreasing since a peak in 1988 and remaining steady at near-1977 levels since 2003. The single most determining factor of death and serious renal sequelae of HUS was undoubtedly the need for dialysis. The rate of serious kidney damage remaining after one year or more was 9%, with another 7.5% of children sustaining lesser renal sequelae. After 10 years or more, the rate of serious damage remained similar, but the rate of observed minor sequelae, such as hypertension and/or proteinuria, rose to 25%.

Our series confirmed classic HUS as a disease of young children, with over half the presentations occurring in the first three years of life. Though the median age of 3.01 years (range 0–14) was somewhat higher than that reported by others [4, 15, 16], it was in line with the mean 3.02 ± 1.51 years as calculated in the Garg meta-analysis [4]. Furthermore, the inclusion of older patients would not have negatively impacted on prognosis, as our entire cohort had a prodrome of diarrhea. In before research, older patients had a worse prognosis because some were diagnosed with atypical HUS, which bears a less favourable outcome [17]. There were slightly more females than males in our cohort, as sometimes [18, 19] but not always [20] reported, though the difference was not statistically significant. 

In Canada, the majority of D^+^ HUS cases are due to VTEC [3, 21]. The incidence of *E. coli* illnesses has dropped substantially since the year 2000, according to Health Canada Statistics (http://dsol-smed.phac-aspc.gc.ca/), as suggested by Proulx and Sockett [14], and as confirmed in our cohort. Yet it is unlikely that *E. coli* O157:H7 virulence and toxicity decreased; the fact that the number of deaths and the percentage of severe cases requiring replacement therapy have not diminished suggests that children in contact with the infectious agent develop the same severe illness as before. However, public education campaigns linking this disease to the consumption of insufficiently cooked ground beef, including the dissemination of guidelines regarding acceptable food preparation, have raised awareness of preventive measures. This is a fine example of the positive influence of the media acting in concert with health agencies to significantly reduce food-borne illness. Nonetheless, the peak observed in 1994-5 is intriguing. This was not associated with any localized outbreak, as cases came from widely dispersed areas. The only explanation we can suggest is that the two summers were strikingly hot with prolonged periods of fair weather creating propitious conditions for outdoor barbecuing of hamburgers, hence the common moniker “hamburger disease.” The fact that the disease was virtually unknown to the public and even to the medical community 35 years ago probably explains the low incidence of recorded HUS cases in the past, when symptoms may not have been diagnosed as such. In contrast, the incidence of classic HUS has not only not declined in countries such as France (http://www.invs.sante.fr/), but also has actually increased. In many European countries where barbecued hamburgers are not as popular as in North America, unpasteurized milk seems to be a major vector for transmission of the disease. For instance, in Italy, raw milk from water buffalos, used in the production of mozzarella cheese, has been shown on occasion to contain VTEC [22]. 

The mortality rate in our study remained steady at approximately 3.6% throughout the 34-year observation period, comparing favourably with the rates in other published reports [4, 14, 20, 23, 24]. These deaths occurred in extremely ill children fraught with multiorgan dysfunction including severe neurological involvement. Although low in absolute numbers, the rate unfortunately has not declined over the years as medical and nursing staff have become more familiar with the disease. Notably, until recently, the only available treatment was supportive. Pharmacologic intervention using newly discovered drugs such as Eculizimab is presently being studied by several investigators, and while some have found that Eculizimab can reduce the severity of the disease in the acute phase [25], others have reported mixed results [26]. 

Requirement for renal replacement therapy in 46% of HUS cases was in line with data reported in comparably large series, where the rate fluctuated widely between 10% and 100%, the average being 65% [4]. Constant surveillance by specialized staff in the intensive care unit during the entire dialysis period doubtless had a positive effect on patient outcome. Nonetheless, patients requiring dialysis developed far more complications than those not requiring dialysis, children with oliguria being known to sustain higher rates of kidney injury [1, 27]. Requirement for dialysis was the sole significant predictor of future renal complications. As many as 33.3% of dialyzed patients showed signs of renal injury at later followup, as compared to only 3% among those not dialyzed. After more than 10 years, 53% of dialyzed patients demonstrated some form of renal impairment as opposed to only 13% of nondialyzed patients. Strikingly, all children who progressed to chronic renal failure, end-stage renal disease, or eventual kidney transplantation did so within 5 years of the acute illness. The 9% rate of renal failure is in line with an incidence of 12% with a low Cr-EDTA-measured GRF (<80 mL/min/1.73 m^2^) in 131 cases in a Scottish study [28], but much lower than the report of Loirat and Fremeaux-Bacchi [6] where, in a cohort of 29 patients, a significant proportion initially considered free of renal involvement ultimately developed renal failure. The overall rate of renal complications after one year or more (17%) was relatively low as compared to a recent large series from Germany and Austria, where as many as 30% were found to have long-term renal sequelae [29]; after 10 years or more, however, our rate approached theirs, at 35%. This confirms Rosales et al.'s suggestion of the need for long-term followup in detecting sequelae [29]. 

The fact that we saw no increase in the incidence of serious complications after a mean followup of 16 years as opposed to 8 years underscores that severe renal complications occur early on, close to or at the time of the acute illness. Adding microalbuminuria to the list of tests to assess renal status proved revealing, however, as it more than doubled the detected findings of kidney injury. Lou-Meda et al. had also reported that screening for microalbuminuria within 6–18 months of the acute illness increased the sensitivity for predicting later renal sequelae by threefold [30]. The clinical importance of testing positive for microalbuminuria is uncertain at this point but can be construed as a harbinger of future complications. These patients may belong to the population subgroup of limited renal reserve, as they showed a decreased GFR following protein loading [31]. This group may benefit from treatment with angiotensin-enzyme inhibitors or angiotensin receptor blockers, as in diabetic nephropathies, as suggested for HUS by Van Dyck et al. [32]. 

A limitation inherent to our study was the loss to follow-up of patients seen more than 10 years previously. As this was a retrospective study design, there was no built-in mechanism for recontacting patients. It has been suggested that those who kept hospital contact for longer periods tended to be the ones with a worse prognosis, those lost to followup tending to be healthier [4]. Indeed, both the incidence of dialyzed patients and the duration of therapy were significantly lower in the subgroup of patients with followup less than one year as compared to the others. It may thus be surmised that the group of 85 participants were sicker than their counterparts lost to followup, as many of the former were identified because they were followed in clinic. Hence, the rate of long-term complications would be overestimated. As concerns the strengths of our study, it must be emphasized that our institution is a referral centre for a large sector of Quebec. As such, our data were trustworthy in sample size and quality. Once discharged from our institution after the acute phase, patients were returned to their referring physician with specific written recommendations for the monitoring of their renal status. Given that children were only 3 years old at the time of the acute illness and that their physician could have redirected these patients to us up until the age of 18, which is the cut-off age for a pediatric institution, we would most probably have seen the majority of children with signs of serious disease. Nonetheless, the results of long-term complications in our series are upward biased by the loss to followup of those with an uneventful course.

In conclusion, survivors of HUS seemingly free of renal damage after the acute phase, even those with minor signs of renal injury such as microalbuminuria or mild proteinuria, should be monitored closely for several decades, especially if dialysis was required. This workup should include measurement of microalbuminuria over a 24-hour period and ambulatory blood pressure monitoring. It would enable the detection of more discreet and later signs of renal involvement. Initially, children should be seen every other year. Once transferred to a family physician and/or adult nephrologist, information concerning possible very late complications should be clearly indicated. It is noteworthy that patients at the time of transfer are teenagers who may often feel invincible and no longer rely on the benefit of close parental supervision. Furthermore, the significant reduction observed in the yearly incidence of D^+^ HUS cases over the last decade makes this report unique in that it suggests that public health organizations and the media can have a positive impact on the prevention of food-borne diseases such as HUS. The threat of an outbreak of an HUS epidemic is always present, however, and public health vigilance needs to be maintained.

## Figures and Tables

**Table 1:** Analysis by length of followup.

Follow-up	<1 year	1 year	*P* value*
(*N* = 325)	(*N* = 126)	(*N* = 199)	
Age at presentation, years	2.77 ± 3.72	3.27 ± 2.72	n.s.
(mean ± SD)			
White blood cell count	16.58 ± 7.6	16.84 ± 6.92	n.s.
(mean ± SD)	×10^9^/L	×10^9^/L	
Patients requiring dialysis, number (%)	44 (34.9%)	102 (51%)	*P* = 0.006
For patients requiring dialysis, average number of dialysis days	6.40 ± 4.83	9.23 ± 6.04	*P* < 0.001
(mean ± SD)			

*P-value determined using Chi-square test.

SD: standard deviation; n.s.: nonsignificant.
